# Evaluation of clinical efficacy, adverse reactions, and safety of PD-1 inhibitors combined with chemotherapy when treating advanced gastric cancer

**DOI:** 10.1186/s12876-023-03011-y

**Published:** 2023-11-01

**Authors:** Xue Huang, Du He, Lin Lai, Jun Chen, Yukun Zhang, Huilin Mao

**Affiliations:** 1 Department of Medical Oncology, Enshi Tujia and Miao Autonomous Prefecture Central Hospital, 158 Wuyang Road, Enshi, 445000 Hubei China; 2 Department of Pediatric Surgery, Enshi Tujia and Miao Autonomous Prefecture Central Hospital, 158 Wuyang Road, Enshi, 445000 Hubei China

**Keywords:** PD-1 inhibitor, Chemotherapy, Advanced gastric cancer, Efficacy, Life quality, Adverse reactions

## Abstract

**Objective:**

This paper aimed to assess the clinical efficacy, adverse reactions, and safety of employing PD-1 inhibitors in conjunction with chemotherapy as a treatment strategy for advanced gastric cancer (GC).

**Methods:**

Ninety patients with advanced GC from January 2020 to December 2021 were divided into the research group (n = 45) and the control group (n = 45). The control group was treated with apatinib and tigio. The study group was treated with PD-1 inhibitor combined with apatinib and tigio. The remission rate (RR), disease control rate (DCR), overall survival (OS), Eastern Oncology Collaborative Group Physical Status Assessment (ECOG-PS) score, EORTCQLQ-C30 (v3.0) score, and incidence of adverse reactions were compared between the two groups.

**Results:**

The research group exhibited improved outcomes in several key metrics relative to the control group. Specifically, the RR, DCR, and OS were notably higher in the research group. Additionally, the ECOG-PS score was significantly reduced, indicating better performance. At a median follow-up of 8.7 months, the research group’s functional and total health scores on the EORTC QLQ-C30 (v3.0) scale had seen significant improvement compared to their initial scores and were also superior to the control group’s scores. Importantly, both groups demonstrated comparable incidence rates for adverse reactions, with no significant difference observed (P > 0.05).

**Conclusion:**

PD-1 inhibitor combined with chemotherapy was more effective when treating patients with advanced GC. It was more beneficial to enhance the patient’s condition, promote survival time, and improve physical status and life quality. In addition, the adverse reactions could be controlled.

**Supplementary Information:**

The online version contains supplementary material available at 10.1186/s12876-023-03011-y.

## Introduction

**Fig. 1:**
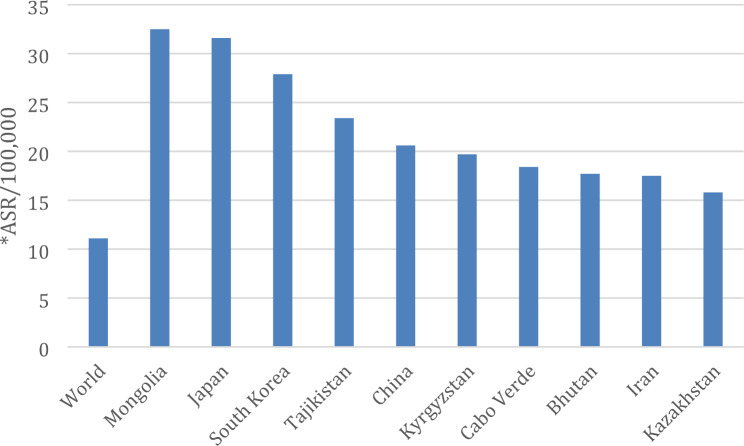
Gastric Cancer global incidence rates 2020 (per 100,000), standardized by age. ASR = age-standardised rates. [Source: World Cancer Research Fund International]

Gastric Cancer (GC) ranks as the fifth most common cancer globally, notable for its high incidence and fatality rates [1]. The 2018 global cancer research data classify GC as the fifth most prevalent malignant tumor overall and the second most common within digestive tract tumors. East Asia, particularly rural areas of China, records exceptionally high GC incidence and mortality rates [2]. In 2015, China reported approximately 403,000 new cases and 291,000 fatalities from GC, making it the second-ranked cancer and the third-highest cause of cancer-related deaths. The 2019 National Cancer Center of China data further confirms these concerning trends, highlighting an unfavorable situation in GC prevention and treatment [3]. Figure 1 shows the global incidence rate of gastric cancer across countries in 2020.

In clinical practice, early Gastric Cancer (GC) symptoms are commonly nondescript, often presenting as only mild epigastric discomfort, and hence, failing to alarm individuals. As a consequence, over 60% of patients receive an initial diagnosis at either locally advanced or metastatic GC stages [4]. The majority of patients are thus detected at a sophisticated stage of GC, which corresponds to unfortunate prognoses with median survival times of merely one year [5]. Research indicates that the 5-year survival rate for stage III GC, even under optimal treatment, is within the range of 15-30%. For stage IV GC patients, this 5-year survival rate distressingly falls to less than 2% [6].

**Fig. 2:**
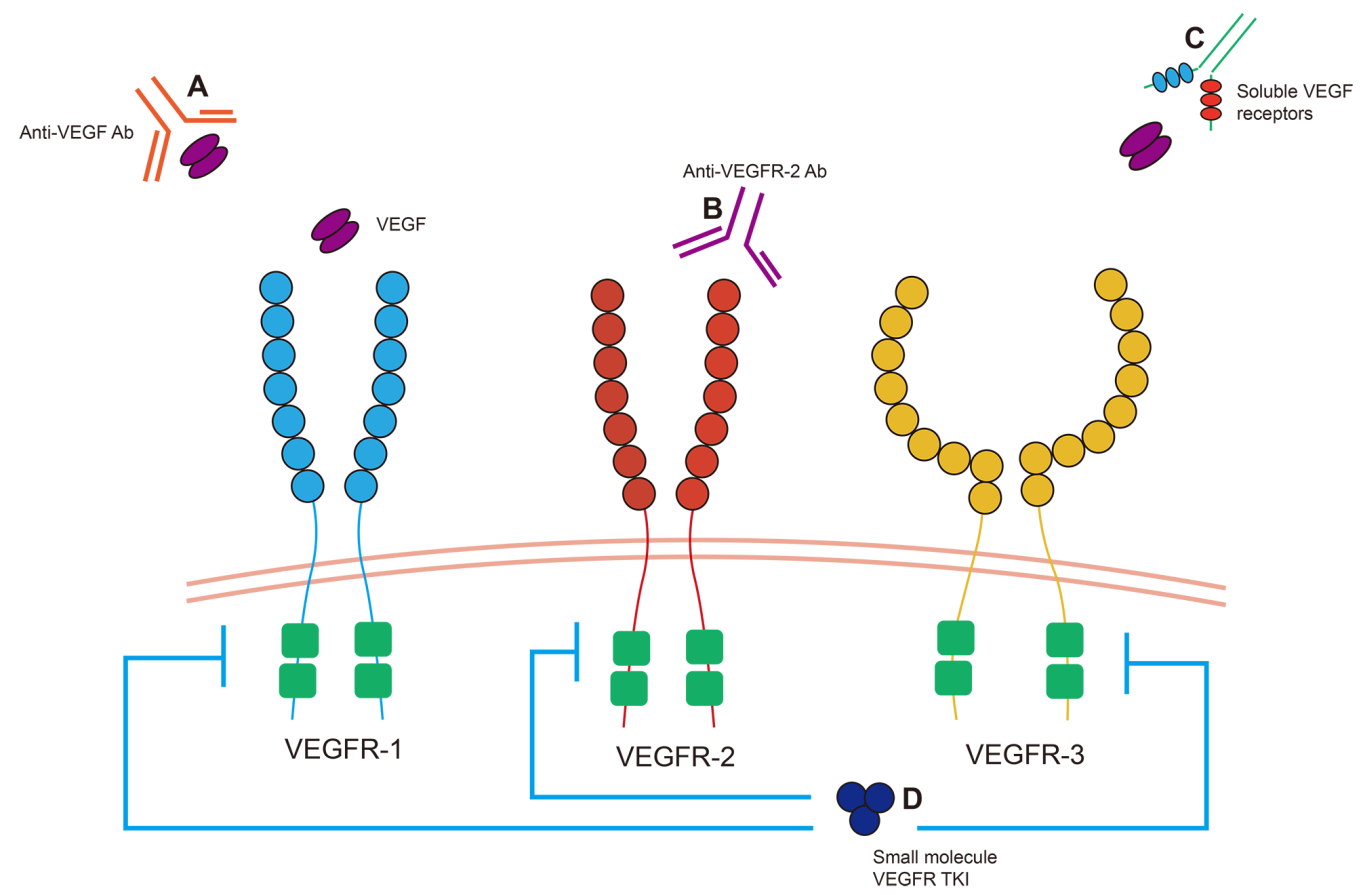
Inhibiting VEGF pathway signaling. Various approaches can be employed to inhibit VEGF pathway signaling, including the use of anti-VEGF antibodies (e.g., bevacizumab), anti-VEGFR-2 antibodies (e.g., ramucirumab), soluble VEGF receptors (e.g., aflibercept), and VEGF receptor tyrosine kinase inhibitors (e.g., sunitinib, sorafenib, regorafenib, apatinib [anti-VEGFR-2 TKi]). These agents target different components of the VEGF pathway to impede its signaling cascade

As a compound preparation based on 5-fluorouracil, Tigio has good bioavailability and anti-tumor activity and can reduce the adverse reactions of the digestive tract and enhance the tolerance of patients on the premise of ensuring the curative effect [7]. Molecular targeted therapy inhibits tumor cell infiltration, proliferation and metastasis through selective blocking of drugs and tumor surface factors. As the TKI of small molecule VEGFR-2, apatinib can combine with ATP in VEGF-2 to interfere with downstream signal transduction, then affect tumor angiogenesis and play an anti-tumor effect [8]. VEGF inhibition can be done in several ways (Fig. 2).

Related studies have shown that apatinib has a good effect in the first-line, second-line and third-line treatment of GC and the patients have low adverse reactions and good tolerance to adverse reactions [9]. However, although targeted drugs can prolong the median OS, the extension is limited [10]. Therefore, the new treatment of advanced GC is worth exploring.

In recent years, programmed cell death ligand 1 (PD-L1) inhibitors can treat advanced solid cancer successfully [11]. Adam et al. found that overexpression of PD-L1 was observed in 65% of GC tissues [12]. The combination of PD-1 and PD-L1 inhibited T cell function, thus inhibiting anti-tumor immune response and promoting tumor growth. The investigation of this mechanism makes blocking PD-1/PD-L1 signal pathway a reasonable target for the treatment of GC patients. Immunotherapy as a hot research field in recent years, the in-depth study of immune checkpoint therapy has also emerged a number of new drugs aimed at this mechanism but also provides a new scheme for the treatment of GC. In 2017, FDA approved Pablizumab for third-line therapy of PD ≥ 1, CPS-positive advanced GC and second-line therapy of any MSI-H/dMMR solid tumor, which opened the era of tumor immunotherapy. Immediately after that, the ICIs provided more choices for patients with GC and more clinical studies were carried out to research the immune combined targeting and immune combined chemotherapy, so as to provide more therapeutic opportunities for patients after the progression of the disease. The Related studies have indicated that early use of immunosuppressants can improve the OS of patients with advanced GC. The KEYNOTE-059 clinical trial developed by Fuchs et al. also indicated that Pembrolizumab, a PD-1 inhibitor, indicated a relatively high response rate and lasting response to PD-L1 positive patients with advanced GC [13]. The KEYNOTE-012 results of a multicenter, open label, phase 1b clinical trial conducted by Muro et al. indicated that Pembrolizumab had controllable toxicity and good anti-tumor activity in PD-L1-positive patients with advanced GC [14]. At present, there are few reports on PD-1 inhibitors combined with chemotherapy when treating advanced GC.

## Methods

### Patients inclusion and exclusion

Patients with advanced GC were collected from January 2020 to December 2021 in Enshi Tujia and Miao Autonomous Prefecture Central Hospital. The diagnostic criteria were based on the 2020 edition of CSCO guidelines for the diagnosis and treatment of GC issued by the Chinese Society of Clinical Oncology, and the staging was based on the 8th edition of the TNM staging system issued by the Union for International Cancer Control (UICC) and the American Joint Committee on Cancer (AJCC) in 2016. The inclusion criteria were: (1) GC or gastroesophageal junction carcinoma confirmed by histology or cytology; (2) abdominal metastasis or other distant metastasis, which cannot be treated by radical operation; (3) ECOG-PS score 0 or 1; (4) initial diagnosis, or received first-line chemotherapy but the tumor progressed; (5) received PD-1 inhibitor therapy combined with chemotherapy; (6) complete clinical data. Exclusion criteria were: (1) patients with autoimmune diseases; (2) there were other tumors and uncontrollable high blood pressure; (3) coagulation dysfunction; (4) therapeutic cycle < 2 cycles; (5) lack of case data or loss of follow-up. Finally, we collected a total of 90 patients with advanced GC from January 2020 to December 2021 in Enshi Tujia and Miao Autonomous Prefecture Central Hospital. This study was approved by the Ethics Committee of Enshi Tujia and Miao Autonomous Prefecture Central Hospital, and written informed consent was obtained from all participants. The patients were followed up until June 31, 2022.

### Treatment strategies

The included patients were randomly divided into the research group (n = 45) and the control group (n = 45). The control group was treated with apatinib and tigio. Apatinib mesylate tablets (Jiangsu Hengrui Pharmaceutical Co., Ltd.): 0.25 g po qd every 21 days as a course of treatment. Tigio capsule (Qilu Pharmaceutical Co., Ltd.): 40 mg po bid d1-d14, every 21 days as a course of treatment, until disease progression, death, or intolerable toxic reactions. The patients were treated with PD-1 inhibitor combined with apatinib and tigio in the research group. The regimen of apatinib and tigio was the same as the control group. The PD-1 inhibitor used in this study was camrelizumab. The specific usage of PD-1 inhibitor was as follows: Camrelizumab for injection (Suzhou Shengdiya Biomedical Co., Ltd.): 200 mg, ivgtt, once every three weeks.

### Outcomes

Remission rate (RR), complete remission (CR), partial remission (PR), and disease control rate (DCR) were studied. In the course of observation, the lesion condition of the patient was evaluated by serum biomarkers and imaging. The curative effect was evaluated according to the RECIST1.1 version. Progression disease (PD) is enlarged by more than 20%, or new lesions appear. Stable disease (SD) is between PR and PD. The drug was evaluated every 3 cycles. The results of the best efficacy evaluation were recorded, and the RR (percentage of patients with RR, CR + PR) and DCR (percentage of patients with DCR, CR + PR + SD) were calculated. Overall survival (OS) was studied and measured in months. The OS refers to the time from the beginning of randomization to death from any cause.

The Eastern Oncology Collaborative Group Physical Status Assessment (ECOG-PS) score in the Eastern American Cancer Cooperation Group was explored. The scoring standard of ECOG-PS scale was 0. One point indicated patients were able to walk freely and engage in light physical activities, including general housework or office work, but not heavy physical activities. 2 points indicated patients could walk freely and take care of themselves, but have lost the ability to work, can get up for not less than half of the day. Three points indicated that patients could only partially care for themselves for more than half the day in bed or in a wheelchair. Four points were bedridden; patients could not take care of themselves. The higher the total score, the worse the physical condition.

EORTC QLQ-C30 (v3.0) score of the life quality scale developed by the European organization for research and treatment of cancer (EORTC) system. The rough score calculation method was that the rough score of each field could be obtained by adding the scores of items included in each field and dividing them by the number of items included. The formula was coarse score RS= (Q1 + Q2+…+Qn) /n. The standardized score transformation formula was “functional area SS=[1-(RS-1)/r] ×100”. Symptom domain and general health symptom domain SS=[(RS-1)/R] ×100. The total scores of functional areas, total health status, and symptoms were 15 ~ 60, 2 ~ 14, and 13 ~ 52, respectively. If the score is higher in the overall health status domain, the better the quality of life; if the score is higher in the symptom domain, the worse the quality of life. The EORTC QLQ-C30 was evaluated before and after treatment (about 6 month after the first treatment).

The incidence of adverse reactions was evaluated. Drug-related adverse reactions were recorded during outpatient follow-up and before admission and were evaluated according to the NCI Common Terminology Criteria for Adverse Events (NCI-CTCAE) recommended by the National Cancer Institute.

### Statistical analysis

The statistical analysis in this study was performed using SPSS 24.0 software. Continuous variables, if normally distributed, were displayed as the mean ± standard deviation; if the distribution was skewed, they were displayed as the median with the interquartile range. Categorical variables were presented in terms of percentages. Statistical differences were determined using the one-way ANOVA test for normally distributed continuous variables, the Kruskal-Wallis test for skewed continuous variables, and the chi-square test for categorical variables. The survival outcomes were illustrated by the Kaplan-Meier curves. P < 0.05 exhibited statistically significant.

## Results

### Patients characteristics

In the research group, there were 21 males and 24 females, aged from 44 to 76 years old with an average age of 60.42 ± 4.17. The primary lesions were GC (n = 33) and gastroesophageal junction carcinoma (n = 12). In the control group, there were 21 males and 24 females, aged from 43 to 75 years old with an average age of 60.53 ± 4.23. The primary lesions were GC (n = 36) and gastroesophageal junction carcinoma (n = 9). There exhibited no significant difference in sex and age between groups (P > 0.05).

### Remission rate and disease control rate

Table 1 summarizes the comparison of RR and DCR between groups. Compared with the control groups, the RR in the research group was significantly higher, while no significant difference was observed in DCR. In the research group, 4.4% achieved CR compared to none in the control group. PR was higher in the research group at 57.8% versus 37.8% in the control. SD was observed in 20.0% of the research participants, while it was 33.3% in the control group. The research group showed a reduced PD rate of 17.8% compared to the control’s 28.9%.

**Table 1:** Comparison of RR and DCR between the two groups

	Research group	Control group	P
Progress			0.080
CR	2 (4.4%)	0 (0.0%)	
PR	26 (57.8%)	17 (37.8%)	
SD	9 (20.0%)	15 (33.3%)	
PD	8 (17.8%)	13 (28.9%)	
RR	28 (62.2%)	17 (37.8%)	0.035
DCR	37 (82.2%)	32 (71.1%)	0.319

CR: complete remission, PR: partial remission, SD: stable disease, PD: progression disease, RR: remission rate, DCR: disease control rate. RR = CR + PR, and DCR = CR + PR + SD

### Overall survival (OS) and the ECOG-PS score

**Fig. 3:**
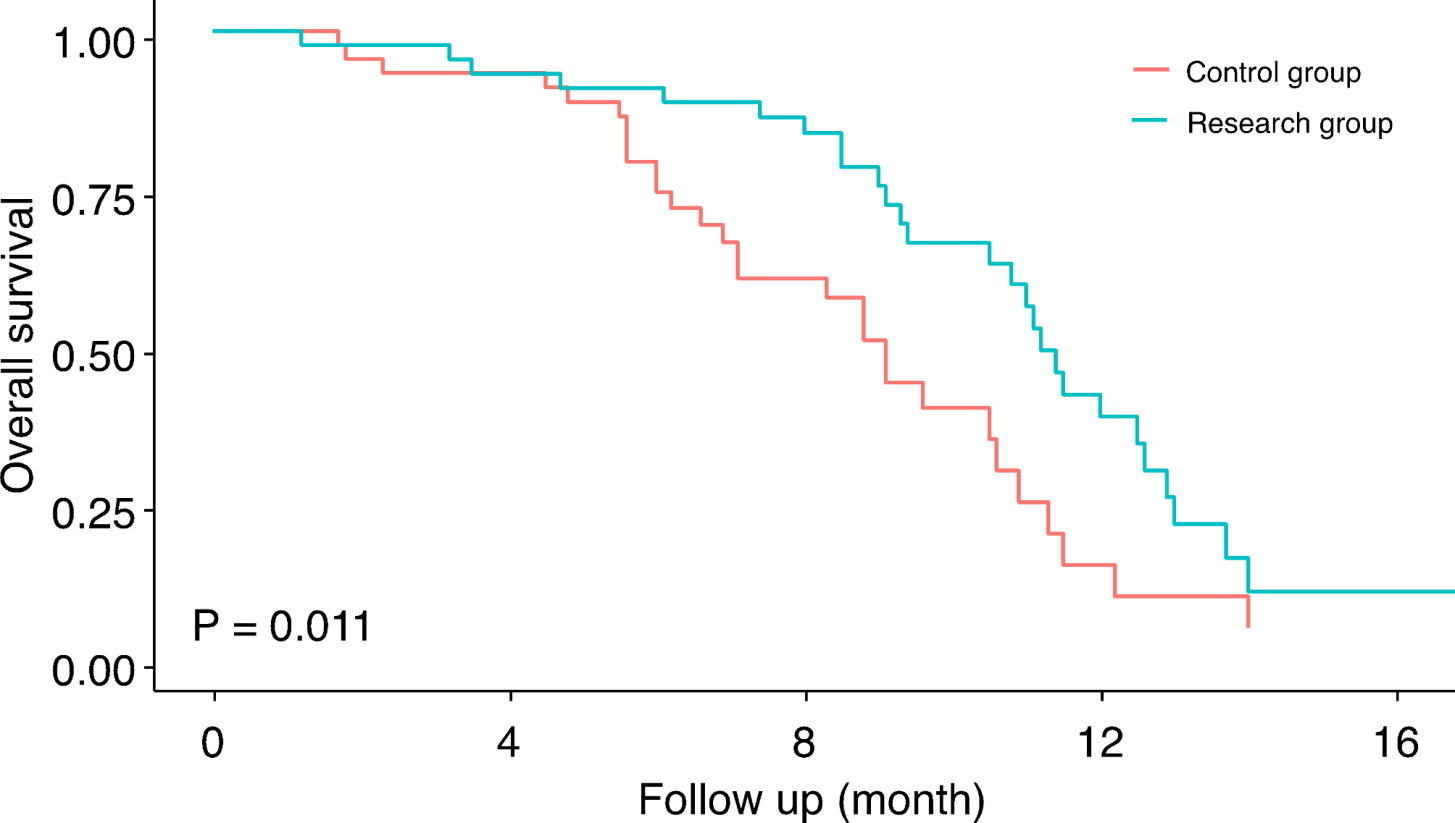
Kaplan-Meier curves for the overall survival of the two groups

After a median follow-up of 8.7 months, the research group demonstrated a significantly longer OS compared to the control group (9.9 ± 2.4 vs. 7.8 ± 2.7 months). Figure 3 shows Kaplan-Meier curves for the OS of the two groups. Regarding the ECOG-PS score, the research group achieved a score of 2.0 ± 0.3, while the control group had a significantly higher score of 2.8 ± 0.5, indicating worse performance.

### EORTC QLQ-C30 (v3.0) score of the study life quality scale

Before treatment, no significant difference was observed in the EORTC QLQ-C30 (v3.0) scores between the research and control groups (P > 0.05), including the symptom score, functional score, and total health score. After treatment, the symptom score of the EORTC QLQ-C30 (v3.0) in the research group was notably lower than the control group (P < 0.05). Also, the score of functional domain and total health domain in the EORTC QLQ-C30 (v3.0) for the research group were significantly higher than the control group (P < 0.05). These comparative findings are clearly delineated in Tables 2 and 3.

**Table 2:** Comparison of life quality scale EORTC QLQ-C30 scores between groups

		Control group	Research group	P
Symptom score	Before treatment	42.4 ± 5.0	42.7 ± 5.2	0.812
	After treatment	29.8 ± 5.0	27.1 ± 3.3	0.003
Functional score	Before treatment	23.3 ± 3.3	23.8 ± 3.0	0.426
	After treatment	32.8 ± 2.5	40.2 ± 3.3	< 0.001
Total health score	Before treatment	3.1 ± 0.3	3.2 ± 0.3	0.490
	After treatment	5.7 ± 1.0	7.1 ± 1.1	< 0.001

**Table 3:** Comparison of life quality scale EORTC QLQ-C30 scores before and after treatment

	Research group			Control group		
	Follow-up	Before treatment	P	Follow-up	Before treatment	P
Symptom	27.1 ± 3.3	42.7 ± 5.2	< 0.001	29.8 ± 5.0	42.4 ± 5.0	< 0.001
Functional	40.2 ± 3.3	23.8 ± 3.0	< 0.001	32.8 ± 2.5	23.3 ± 3.3	< 0.001
Total	7.1 ± 1.1	3.2 ± 0.3	< 0.001	5.70 ± 1.0	3.1 ± 0.3	< 0.001

### Incidence of adverse reactions

There were 21 cases of adverse reactions in the research group. We observed no significant difference in the incidence of adverse reactions between groups (P > 0.05). The comparison of the incidence of adverse reactions between groups is shown in Table 4.

**Table 4:** Comparison of the incidence of adverse reactions

	Research group	Control group	P
Myelosuppression (case, %)	6 (13.3%)	7 (15.6%)	1.000
Hypothyroidism (case, %)	5 (11.1%)	7 (15.6%)	0.756
High blood pressure (case, %)	7 (15.6%)	5 (11.1%)	0.756
Dermatotoxicity (case, %)	6 (13.3%)	5 (11.1%)	1.000

## Discussion

GC is a prominent malignant tumor in the upper digestive tract [15]. Its early symptoms are often understated, and less obvious than common digestive system diseases. Although surgery remains the primary treatment [16], many patients are diagnosed at an advanced stage due to the inconspicuous characteristics of early GC [17, 18]. Currently, the standard treatment for advanced GC is chemotherapy-based comprehensive therapy [19]. Despite this, it proves insufficient to improve the prognosis of advanced GC patients. Fluorouracil was the first drug found effective against GC, forming the basis for various 5-fluorouracil-based regimens such as FAM [20]. Additionally, 5-fluorouracil and platinum-based regimens like CF or ECF came into use. Nonetheless, these two types of drugs often lead to drug resistance and poor efficacy due to their widespread usage [21]. The ongoing discovery and research of drugs like paclitaxel and irinotecan offer patients more options post first-line treatment failure [22]. A meta-analysis by Wei Suxian revealed mPFS5.2-9.0 months and mOS8.3-14.59 months for the three-drug combination regimen [23]. Although this regimen was ineffective at reducing death risk, it extended Progression-Free Survival (PFS) in patients. Today, first-line chemotherapy for advanced GC patients still relies on 5-fluorouracil and platinum while failed first-line treatments primarily use paclitaxel, irinotecan, docetaxel, and albumin-bound paclitaxel.

Because of the heterogeneity of GC, the grade of malignancy of advanced GC is high, and the benefit of clinical treatment is low. Single-drug treatment is prone to drug resistance, resulting in rapid disease development and high mortality. The patient’s condition progressed after standardized comprehensive treatment, and how to treat it after the progress is an important problem encountered in the clinic. The treatment of GC is developing in the direction of chemotherapy, targeted combined chemotherapy, immune combined targeting, and chemotherapy. The application of immunotherapy in GC brings hope for treating advanced GC. A few studies have indicated that PD-L1 is highly expressed in GC. Human-activated cytotoxic T lymphocytes can express PD-1. PD-1 binds to PD-L1, which leads to CTL apoptosis and suppresses immune response [24]. Immune checkpoint is equivalent to the negative regulation of immune regulation, which prevents and treats the damage of immune cells in the average human body. Tumor cells use this negative regulation to evade human immune surveillance by interfering with CTLA-4, PD-1, and PDL-1 immune checkpoints [24]. ICIs activate the human immune system, identify tumor cells and kill them by specifically binding to the immune checkpoints on the surface of tumor cells. Some studies have indicated that chemotherapy and PD-1 inhibitors have a synergistic anti-tumor effect. Chemotherapy can enhance the immunogenicity of tumor cells, promote antigen presentation and eliminate immunosuppressive cells in host cells, thus enhancing tumor immune response []. According to the analysis of the safety and efficacy of KEYNOTE-061 when treating advanced GC with palivizumab, it was concluded that OS could be prolonged in patients with GC with MSI-H or PD-L1CPS ≥ 1, so it was approved as a specific type of advanced GC with MSI-H or PD-L1CPS ≥ 1 [26]. The study of Navuliu monoclonal antibody showed that it could benefit both MSI and PD-L1 status in patients with GC [27]. Immunotherapy can promote tumor cell necrosis and release new antigens, promote immunosuppressive cell necrosis, improve the inhibition of tumor microenvironment on immune function, and reactivate the immune system [28]. Therefore, immunotherapy combined with chemotherapy is essential to overcome drug resistance and may benefit patients with advanced GC.

The results of this study indicated that the RR and DCR of patients after PD-1 inhibitor combined with chemotherapy were higher than chemotherapy alone. The value of OS was remarkably longer than chemotherapy alone. ECOG-PS score was remarkably lower than chemotherapy alone. After treatment, the symptom areas in the EORTCQLQ-C30 (v3.0) scale were remarkably lower than those before treatment, and they were remarkably lower than those treated with chemotherapy alone. By the 6-month point, scores related to the functional domain and total health domain in the EORTC QLQ-C30 (v3.0) scale had significantly improved compared to pre-treatment or chemotherapy alone. There was no notable difference in the adversarial reaction incidence between the two therapeutic strategies. Symptomatic treatment improved while the combined treatment method was well tolerated. Overall, it was established that the PD-1 inhibitor combined with chemotherapy is more efficacious in treating patients with advanced GC.

Moreover, it is more beneficial to enhance the patient’s condition, survival time, physical condition, and life quality and the adverse reactions can be controlled. This is mainly because PD-1 inhibitors can enhance the sensitivity of chemotherapy by enhancing the anti-tumor immune response to improve the effectiveness of anticancer treatment, enhance RR, DCR, and OS, and promote physical status and life quality [29, 30]. In the KEYNOTE-059 clinical trial, the effective rate of PD-1 inhibitor Pablizumab combined with chemotherapy (5-MFU + cisplatin or capecitabine) for first-line advanced gastroesophageal junction cancer was 60% (95% CI: 38.7-78.9%) [31]. In the KENOTE-062 clinical trial, RR was 48.6% (CPS ≥ 1) and 52.5% (CPS ≥ 10) in patients with advanced GC who received first-line chemotherapy with palivizumab combined with cisplatin and 5-Fu (or capecitabine), PFS can reach 6.9 months (CPS ≥ 1) and 5.7 months (CPS ≥ 10) [32]. In the Japanese KENOTE-659 clinical trial, Pablizumab combined with oxaliplatin regimen first-line chemotherapy for advanced GC, the RR was 72.2%, and the PFS was 9.4 months [33]. In the first part of the ATTRACTION-4 clinical trial, the RR of patients with advanced GC was 57.1% in first-line chemotherapy with Navulizumab combined with tigio and oxaliplatin. The PFS was 9.7 months, and the patients receiving nivolumab combined with capecitabine and oxaliplatin chemotherapy achieved an RR of 6.5% and a PFS of 10.6 months [34]. In terms of drug safety, the adverse reactions in this study were consistent with the adverse reactions of traditional chemotherapy. However, PD-1 inhibitors were reported in previous clinical trials, and there were no unexpected adverse reactions [35]. The same idea can be found in the study put forward by Zhao Z [36]. They have applied new methods in the study, and the conclusions drawn can also give some support to this study. The same idea can be found in the study put forward by Gao G et al. [37]. They have applied new methods in the study, and the conclusions drawn can also give some support to this study.

## Conclusion

In conclusion, when treating patients with advanced GC, combining PD-1 inhibitors with chemotherapy proves more effective. This combination not only enhances patients’ conditions and survival time but also improves their physical status and quality of life. Of note, the adverse reactions are controllable. Despite presenting meaningful clinical findings, our study does possess certain limitations. The small sample size restrains the study’s overall quality. Also, as a single-center study, our findings may exhibit a bias, potentially diverging from the results of large-scale multicenter studies conducted by other academic institutions. Despite these limitations, our research holds clinical significance and calls for subsequent, more profound investigations in the future.

### Electronic supplementary material

Below is the link to the electronic supplementary material.

Supplementary Material 1

## Data Availability

The datasets generated during and/or analysed during the current study are not publicly available due to institutional policies but are available from the corresponding author on reasonable request.
