# Supplementary material for: Evaluation of clinical efficacy, adverse reactions, and safety of PD-1 inhibitors combined with chemotherapy when treating advanced gastric cancer
Source: BMC Gastroenterol. 2023 Nov 1;23:374. doi: 10.1186/s12876-023-03011-y (PMC10619224; doi:10.1186/s12876-023-03011-y)
Supplement: Supplementary file 1 — Supplementary Material 1 [file 12876_2023_3011_MOESM1_ESM.pdf]

伦理简易审查意见函

审查日期： 2023年9月11日

项目名称	Evaluation of clinical efficacy, adverse reactions and safety of PD-1 inhibitors combined with chemotherapy when treating advanced gastric cancer		
项目类别	论文	项目来源	
组长单位	无		
审查方式	简易审查	审查类别	初始审查
主要研究者	黄雪	主要研究者科室	肿瘤一科
审查文件及版本号	递交信、伦理初始审查申请表、任务书、研究方案（版本号V1.0;版本日期2021年10月15日）、知情同意书（版本号V1.0;版本日期2021年10月15日）、风险评估与防控预案（版本号V1.0;版本日期2021年10月15日）、主要研究者简历、主要参与研究者简历、主要研究者责任声明		
评审意见	审查决定：		
	恩施土家族苗族自治州中心医院医学伦理委员会主任委员：张[illegible]		签发日期：2023年9月11日
年度/定期跟踪审查频率	3个月☐ 6个有☐ 12个月☑不调整原有频率☐		
有效期：			
